# *T*ranexamic *a*cid vs. em*b*olization of the meningeal artery as an adjunctive therapeutic regime to reduce the recurrence rate after *s*urgical relief of *c*hronic subdural hemat*o*mas (TABASCO)—a randomized controlled trial

**DOI:** 10.1186/s13063-025-08888-6

**Published:** 2025-06-14

**Authors:** Magnus Scheer, Vanessa Beuchel, Uwe Max Mauer, Klaus Efinger, Chris Schulz

**Affiliations:** 1 Department of Neurosurgery, German Armed Forces Hospital Ulm, Oberer Eselsberg 40, 89081 Ulm, Germany; 2 Department of Neurosurgery, German Armed Forces Hospital Westerstede, Lange Str. 38, 26655 Westerstede, Germany; 3 Department of Radiology and Neuroradiology, German Armed Forces Hospital Ulm, Oberer Eselsberg 40, 89081 Ulm, Germany

**Keywords:** Mild traumatic brain injury, Chronic subdural hematoma, Tranexamic acid, Embolization, Surgery, Endovascular, Middle meningeal artery, Randomized controlled trial

## Abstract

**Background:**

Chronic subdural hematoma is a persistent, bloody to serous fluid retention in the subdural space between the dura mater and arachnoid mater, usually caused by an initial trauma, and is one of the most common traumatic intracranial hemorrhages in western industrialized nations. In the event of a compressive effect on the brain, the hematoma is usually relieved by means of a burr hole trephination. In view of the high postoperative recurrence rate, conservative treatment methods have been investigated both as a competitor to surgery alone in cases where the indication for surgery is debatable and as a supportive therapy for surgical hematoma relief. Studies on embolization of the middle meningeal artery during surgery as well as studies on postoperative drug therapy using tranexamic acid have shown the most promise. However, there is currently a lack of studies that randomly compare the effectiveness of these two perioperative treatment strategies regarding to their efficacy in avoiding revision surgery and the safety of the respective procedure.

**Methods:**

TABASCO is a prospective, randomized, two-arm, multicenter, clinical trial designed to determine whether postoperative treatment of chronic subdural hematomas using adjuvant drug therapy with tranexamic acid (test group) is equivalent to postoperative embolization of the arteria meningea media (control group) in terms of postoperative volume reduction of the hematoma and the need for revision surgery of CSDH. Patients over 18 years of age who have undergone surgery for CSDH for the first time no more than 24 h before inclusion in the study will be randomized 1:1 to the test group or control group. The primary endpoint is the postoperative volume decrease of a primarily surgically relieved CSDH quantitatively and regarding to the time course on the affected side in a study period of 3 months postoperatively. The secondary endpoint of this study is to investigate the extent to which the rate of necessary revision surgery can be influenced by the adjuvant therapy procedures over the course of 3 months. The tertiary endpoint is the neurological outcome of the patients included in the study and assigned to the different treatment arms after a total follow-up period of 3 months as well as the complication rate of the adjuvant procedures used. Assuming a risk difference of 8% for rebleeding and surgical revision, with an applied power of 80%, 276 patients (138 per group) will be included in this study.

**Discussion:**

The TABASCO study will provide clinical evidence as to whether embolization of the middle meningeal artery in addition to surgery is comparable to postoperative drug therapy using tranexamic acid as an adjuvant treatment method for operated chronic subdural hematomas in terms of hematoma volume reduction, revision rate and safety of the procedures.

**Trial registration:**

German Clinical Trials Registry (Deutsches Register Klinischer Studien (DRKS)) DRKS00033515. Registered on 05 Feb 2024.

**Supplementary Information:**

The online version contains supplementary material available at 10.1186/s13063-025-08888-6.

## Introduction

### Background and rationale {6a}

Chronic subdural hematoma (CSDH) is a persistent, bloody to serous fluid retention in the subdural space between the dura mater and arachnoid mater, usually caused by an initial trauma, and is one of the most common traumatic intracranial hemorrhages in western industrialized nations [23]. The incidence rate is described between 1.7 and 20.6 per 100,000 inhabitants, whereby there is a clear age dependency. The incidence rises with increasing age and the demographic change with an elderly population as a whole on the other hand [6]. In this context, Finnish studies have shown a doubling of the incidence from 8.2 to 17.6 per 100,000 inhabitants over the last 25 years [17].

The pathophysiology leading to the development of CSDH is yet not fully understood. Originally, it was assumed that initial traumatic bleeding was caused by tears in the intracranial bridging veins, which led to additional fluid absorption in the subdural space through osmosis and thus to liquefaction and progression in size of the hematoma. However, current studies show a much more complicated pathogenesis. In contrast to acute SDH, which is caused by the rupture of bridging veins or cortical vessels, the cause of CSDH also lies in injury to the so-called dural border cell layer. This is located between the dura mater and the arachnoid mater and consists of a group of highly specialized connective tissue cells. If these cells are injured, local inflammatory reactions are set in motion. They lead to increased fibrinolysis and angiogenesis, through which the renewed healing of this layer is sought. As part of this process, an inner and outer membrane are formed. The inner membrane consists of collagen and fibroblasts and lies against the arachnoid, while the outer membrane merges into the dura mater. It contains various cells and mediators that maintain the inflammatory process. Within this framework, not only the membranes continuously are formed, but membrane septa can also develop within the CSDH. In addition, new (pathological) capillaries are formed, which have very thin walls. The fragile capillaries within the outer membrane rupture easily, which leads to renewed microbleeds into the interior of the membrane, while at the same time the plasminogen-activated hyperfibrinolysis of the membrane contents as part of the acute phase reaction leads to the prevention of regular clot formation, which in turn promotes persistent microbleeds [8]. The bleeding again stimulates the inflammatory reaction and a vicious circle of cell proliferation, angiogenesis, hyperfibrinolysis and bleeding through rupture of newly formed vessels develops [3, 17, 20]. This leads to a continuous progression of the hematoma size.

The gradual increase in volume of the hematoma can lead to displacement or a compressive effect on the adjacent brain tissue and thus to the development of neurological symptoms with the development of headaches, central motor deficits, epileptic seizures and reduced vigilance and even coma.

Where narrow, asymptomatic, chronic subdural hematomas can still be treated conservatively as part of a wait and watch or wait and scan strategy [17] with regular imaging follow-up and the interruption of any additional antiplatelet or anticoagulant therapy, hematomas with a proven compressive effect from a thickness of around 10 mm and a midline displacement of 7 mm [17] and the associated clinical symptoms must be surgically relieved.

Various techniques can be used (depending on the localization, the imaging of the CSDH and the space-occupying effect). These include burr hole trepanation as well as smaller and larger craniotomies, irrigation of the hematoma cavity and placement of a subdural drainage system [13]. Regardless of the type of surgery performed, however, a relatively high recurrence rate of 2.3–38.7% has been shown with the need for revision surgery [15, 18].

In view of the high postoperative recurrence rate, conservative treatment methods have been investigated both as a competitor to surgery alone in cases where the indication for surgery is debatable [17] and as a supportive therapy for surgical hematoma relief. Studies on embolization of the middle meningeal artery (MMA) [21] during surgery as well as studies on postoperative drug therapy using tranexamic acid were the most promising.

It was shown that postoperative drug therapy with tranexamic acid, which is a specific antifibrinolytic drug that inactivates plasminogen and thus inhibits hyperfibrinolysis [12] in order to prevent a renewed increase in the size of the CSDH by preventing microbleeds, reduced the recurrence rate and thus also the re-operation rate compared to surgical treatment of CSDH alone [2, 6, 10, 13, 14, 24, 25, 28]. In addition, several studies have shown that interventional embolization of the arteria meningea media, which is known to supply the dura mater arterially, and thus the occlusion of distal microvessels of this vessel also reduces the active processes of cell proliferation, angiogenesis and secretion, which cause the recurrence of CSDH [1, 7, 16, 19, 22].

However, there is currently a lack of studies that randomly compare the effectiveness of these two perioperative treatment strategies regarding to their efficacy in avoiding revision surgery and the safety of the respective procedure.

### Objectives {7}

#### Primary objective

The primary aim of this study is to investigate how the postoperative volume decrease of a primarily surgically relieved CSDH can be influenced quantitatively and regarding to the time course by a postoperative adjuvant drug therapy with tranexamic acid in comparison to a postoperative interventional embolization of the MMA on the affected side in a study period of 3 months postoperatively.

#### Secondary objective

The secondary objective of this study is to investigate to what extent the rate of necessary revision interventions of a primarily surgically relieved CSDH can be influenced by postoperative adjuvant drug therapy with tranexamic acid compared to postoperative interventional embolization of the middle meningeal artery on the affected side over the course of 3 months postoperatively.

#### Tertiary objectives

The tertiary objective of this study is the presentation and evaluation of.

- The constellation of findings that led to the indication for revision surgery in patients included in the study,
- The neurological outcome of the patients included in the study and assigned to the different treatment arms after a total follow-up period of 3 months,
- Complications related to the interventional procedure used, and
- Complications and side effects due to the use of tranexamic acid.

### Trial design {8}

TABASCO is an investigator-initiated, multi-center, randomized controlled trial testing whether postoperative treatment of chronic subdural hematomas using adjuvant drug therapy with tranexamic acid (test group) is equivalent to postoperative embolization of the MMA (control group) in terms of postoperative volume reduction of the hematoma and the need for revision surgery of CSDH within a follow-up period of 3 months. Eligible patients will be allocated in a 1:1 ratio to either the test or control group.

## Methods: participants, interventions, and outcomes

### Study setting {9}

In all participating centers, after diagnostic confirmation of a unilateral chronic subdural hematoma and an associated indication for surgical relief of the hematoma, it is checked within 24 h after surgery whether they are eligible for participation in the study. Screening for inclusion in the study is carried out. After informed consent has been obtained, the patient is randomly assigned to either the test or the trial group. With randomization to the test group, adjuvant drug therapy with tranexamic acid is started. If assigned to the control group, embolization of the middle meningeal artery on the diseased side will be performed within 72 h postoperatively. The study participants are monitored according to the follow-up schedule (Table 1). All clinical investigators in all participating centers have renowned expertise in neurosurgery, neuroradiology, and endovascular interventions. Structural and process quality in terms of state-of-the-art radiological imaging, surgery, critical care, and management of complications are provided.

**Table 1:**
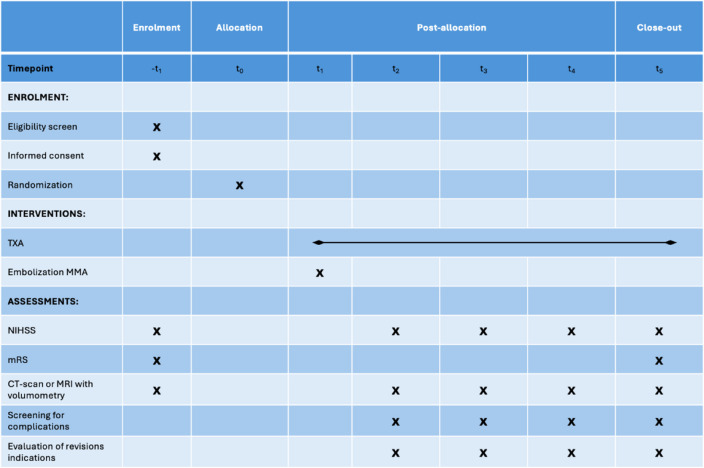
Study schedule

*− t*_1_*:* Recruitment and information, *t*_0_: randomization,* t*_1_: postoperative period of 72 h in which the procedure with embolization of the MMA is performed or drug therapy with TXA is started, *t*_2_: follow-up 1 week postoperative,* t*_3_: follow-up: month postoperative (± 1 week), *t*_4_: follow-up: 2 months postoperative (± 1 week),* t*_5_: follow-up: 3 months postoperative (± 1 week) and close out

### Eligibility criteria {10}

#### Inclusion criteria

1. Patients who underwent surgery for unilateral CSDH for the first time no more than 24 h before inclusion in the study
2. Age ≥ 18 years
3. Written informed consent to participate in the study by the patient or a legal representative

#### Exclusion criteria

1. Conservatively treated CSDH
2. Age ≤ 18 years
3. Bilateral CSDH
4. Pregnancy and breastfeeding
5. Radiological evidence of an acute subdural hematoma, subarachnoid hemorrhage, epidural- or intracerebral hematoma
6. Structural causes of subdural hemorrhages, e.g., arachnoid cysts, cortical vascular malformations
7. Lack of inform consent
8. Inability to perform angiography/embolization within 72 h with simultaneous contraindications for taking tranexamic acid (inability to crossover)
9. Previous history of burr hole trepanation, craniotomies, or craniectomies for other reasons

### Who will take informed consent? {26a}

Informed consent to participate in the trial will be obtained by the patients’ treating physician. Patients or their authorized surrogates will be given ample of time to ask questions and are requested to provide written informed consent within 24 h. The informed consent form will be signed by one of the researchers and the trial participant, or authorized surrogate if the trial participant has a decreased level of consciousness. In the latter case, informed consent will be obtained in the second instance if the trial participant becomes mentally competent during the study follow-up.

In the event of randomization to the control group, a specialist in neuroradiology will obtain separate informed consent about the embolization procedure.

### Additional consent provisions for collection and use of participant data and biological specimens {26b}

Together with informed consent for participation in the trial, additional consent is asked for reuse of the pseudonymised collected data for future research questions. For this study, no biological specimens will be collected.

## Interventions

### Explanation for the choice of comparators {6b}

Patients will be randomized into two groups, tranexamic acid vs. embolization with a 1:1 allocation after surgical relief of a chronic subdural hematoma. In the tranexamic acid group, postoperative adjuvant administration of 1500 mg tranexamic acid per day is performed over a period of 3 months; in the embolization group, embolization of the MMA on the affected side is performed 72 h postoperatively. The efficacy of both treatment methods in the adjuvant treatment of chronic subdural hematomas requiring surgery has been confirmed. However, there is no evidence-based, standardized postoperative treatment regime to reduce the incidence of recurrence in chronic subdural hematomas.

### Intervention description {11a}

1. Test group:

Patients who are randomized into the test group after verification of the inclusion criteria and consent for inclusion in the study will be treated adjuvantly after successful surgical relief (burr hole trepanation and/or any surgical technique) of CSDH within 48 h over a period of 3 months using a medication regimen of a total of 1500 mg tranexamic acid per day, divided into 2 or 3 daily doses, per os, over a period of 3 months.

The following contraindications to take this medication must be checked as part of the study inclusion:

- Hypersensitivity to the active substance
- Acute thrombosis or thromboembolic diseases, such as deep vein thrombosis, pulmonary embolism and cerebral venous thrombosis
- Condition following a history of venous or arterial thrombosis.
- Hyperfibrinolytic conditions in the context of a consumption coagulopathy
- Severe renal insufficiency/renal dysfunction
- History of seizures
- Color vision deficiency
- Massive bleeding from the upper urinary tract (especially in hemophilia) [5]

(in the event of randomization into the test group with existing contraindications of taking tranexamic acid, *crossover into the control group* is possible).

2. Control group:

Patients who were randomized into the control group after checking the inclusion criteria and consenting to inclusion in the study after randomization are treated within 72 h postoperatively after successful surgical relief of the CSDH by means of endovascular embolization of the MMA on the affected side.

Embolization is performed using a microcatheter, which is inserted minimally invasive under sedation or intubation anesthesia via a transfemoral approach (alternatively also transradial) into the middle meningeal artery on the affected side. The target vessels are identified using digital subtraction angiography (DSA). This means increased radiation exposure for the patients assigned to the test group. The MMA originates in the infratemporal fossa from the maxillar artery, a branch of the external carotid artery. It enters the base of the skull through the foramen spinosum and gives off several branches to the dura mater from the point of entry. The middle meningeal artery can have different variations. Anastomoses with other branches of the external carotid artery and the internal carotid artery are possible. In particular, the variant in which the middle meningeal artery arises from the ophthalmic artery, which is probably more common than average in patients with CSDH, is an absolute contraindication for embolization, as there is an increased risk of central retinal artery occlusion with subsequent blindness [20]. In this case, crossover to the test group is possible.

The vasa nervorum of the facial nerve, the greater petrosal nerve and the trigeminal nerve usually originate from the middle meningeal artery shortly after passing through the foramen spinosum. Corresponding neurological deficits are therefore also possible here in the event of vascular occlusion. These anastomoses can usually be visualized well by angiography. To avoid complications due to incorrect embolization, care is taken not to embolize the proximal section of the middle meningeal artery [20].

A liquid embolizate like ONYX® or micro-electric coils can optionally be used to embolize the MMA.

### Criteria for discontinuing or modifying allocated interventions {11b}

Patients may withdraw their consent at any time without providing a reason and thus terminate their participation in the study prematurely. Withdrawal from the study and reasons, if known, will be documented. Study treatment stops if a surgical treatment for the CSDH is necessary during follow-up, in case of subsequent occurrence of an exclusion criterion or in occurrence of treatment-related complications during treatment. Crossover to the other study arm is not possible after the start of treatment in the respective study arm. Moreover, the principal investigator is entitled to terminate the study prematurely if new scientific findings in favor of one or the other treatment violate the equipoise principle.

### Strategies to improve adherence to interventions {11c}

1. Test group:

Treatment as part of the study takes place within 3 months of inclusion in the study. Follow-up visits will take place after 1, 4, 8, and 12 weeks. Upon enrollment in the study, the participant will receive instructions on how to take the medication, including when to take it, how to store it, the importance of taking the capsules as a whole and what to do in the event of a missed dose. The importance of adhering to the study protocol is also discussed.

At the follow-up visits, compliance with the treatment is monitored by asking whether the medication was taken regularly at the prescribed times, at what times the capsules were taken and whether the participant experienced any side effects. Study participants are asked to contact one of the researchers if they have any questions about the study medication during the treatment period. Medication monitoring will be performed by one of the researchers or by the research nurse who will also perform the follow-up measurements. The reason for any non-compliance will be recorded on the designated case report form.

2. Control group:

There is no possibility to influence patients’ adherence in that study group to the intervention. Embolization of the MMA constitutes the only study intervention, which is solely performed by the clinical investigators.

### Relevant concomitant care permitted or prohibited during the trial {11 d}

Except for the study intervention, patients in both groups are treated according to the currently established standard of care at the participating trial centers. Any concomitant care as part of routine clinical practice is permitted.

### Provisions for post-trial care {30}

Parallel to study participation, all study participants receive standard care. This includes extended follow-up after study participation has ended, if necessary. A study insurance for trial-participants is not provided.

### Outcomes {12}

#### Primary outcome

The primary element to be investigated in this study is the postoperative volume development of a primarily surgically relieved unilateral CSDH over the course of 3 months postoperatively. For diagnostic purposes and documentation, cranial cross-sectional imaging using native cMRI or CT-scan and subsequent volumetry to visualize the residual hematoma volume in milliliters will be performed at the planned follow-up time points, 1 day postoperatively, 1 week postoperatively, 1 month postoperatively, 2 months postoperatively and 3 months postoperatively. The volumetry will be performed by a radiologist who is not involved in the study. Although he is not informed about the group assignment of each patient, he is not blinded because the group assignment can easily be identified from the radiological evidence of the embolism material in the imaging. All findings are considered in relation to each other and in relation to the initial findings before the operation. The planned examination intervals correspond to the current standard of care for postoperative imaging follow-up after CSDH surgery and reflect the procedure used in comparable studies [27].

#### Secondary outcome

To answer the question specified in the secondary objective, the need for revision surgery on the primary side of patients included in the study is documented depending on the study arm as well as the time interval to the primary procedure.

#### Tertiary outcome

In addition to answering how the postoperative volumes of a primarily surgical relieved CSDH can be influenced by the administration of TXT compared to embolization of the MMA and how high the re-operation rate per treatment arm is, the aim is to show which constellation of symptoms lead to the indication for revision-operation in each case. For this purpose, the hematoma volumes imaged at the follow-up periods will be correlated with the neurological deficits recorded at the follow-up times according to the NIHSS score. Furthermore, the neurological outcome at the end of the study at the 3-month follow-up will be assessed using the modified Rankin Scale and the NIHSS, the results will be compared with the baseline values before surgical hematoma relief.

The National Institutes of Health Stroke Scale (NIHSS) is a scoring system from the field of neurology and was originally used for the early detection and monitoring of stoke progression. The NIHSS score covers the following areas: Level of consciousness, eye movements, integrity of visual fields, facial movements, arm and leg muscle strength, sensation, coordination, speech, language and neglect. Each impairment is rated on an ordinal scale of 0 to 2, 0 to 3, or 0 to 4. The scores are summed up to give a total score of 0 to 42 (the higher the score, the more severe the neurological deficit) [9]. In addition to the diagnosis and follow-up of ischemic strokes, the NIHSS represents an evaluation of the core symptoms of a chronic subdural hematoma that is accepted in the current literature, both in the context of acute diagnosis and in the follow-up situation.

The modified Rankin Scale measures the degree of disability or dependence in the daily activities of people who have suffered a stroke or other causes of neurological disability. The following scaling is used [26].

0: No symptoms.

1: No relevant impairment. Can carry out everyday activities without restriction despite minor neurological deficits.

2: Slight impairment. Can take care of themselves without help but is restricted in everyday life.

3: Moderately severe impairment. Needs help in everyday life but can walk without assistance or with aids (walking stick, rollator).

4: Severe impairments. Needs help with personal hygiene, cannot walk without assistance.

5: Severe disabilities. Bedridden, incontinent, requires constant nursing assistance.

In addition, complications in relation to the interventional procedure and complications and side effects due to the use of tranexamic acid will be documented.

### Participant timeline {13}

**Fig. 1:**
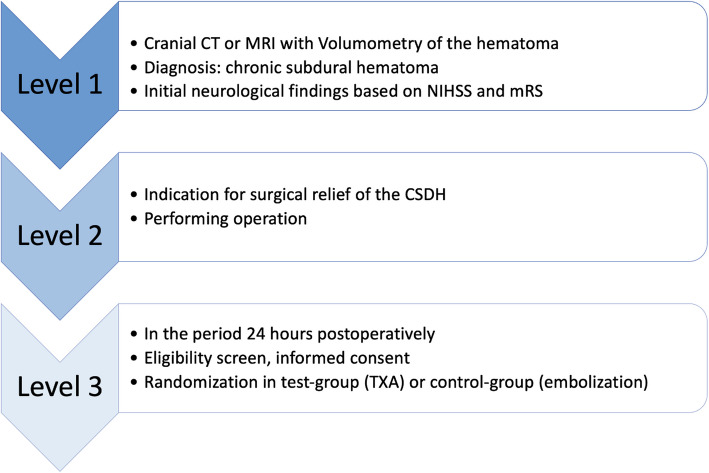
Steps that have to be carried out, before potential participations can be enrolled in the study

According to the study protocol, patients must have undergone surgery for a chronic subdural hematoma within 24 h prior to study inclusion. In this context, the following steps must be carried out before inclusion in the study (Fig. 1). The schedule for participants from study entry is shown in Table 1.

### Sample size {14}

In order to estimate the required sample size, the volume change of a primary operated CSDH under the adjuvant therapy forms to be investigated is analogized with the recurrence rates of primary operated and adjuvant embolized or adjuvant drug-treated CSDH in the literature with regard to the target value defined within the primary objective. For example, the MEMBRANE study, which is currently being recruited, assumes a recurrence rate of 10% when combined surgical and interventional therapy is used [11]. In comparison, the work of Hibahashi et al. [27] puts the recurrence rate of CSDH treated with a combination of surgery and medication at around 2%. Based on this, using the *z*-test for independent samples and an error of the first kind (*α* = 0.05), a sample size of 276 patients (138 per group) is required to achieve a power > 0.8 to detect a difference of 8%.

### Recruitment {15}

All physicians at participating centers treating CSDH patients will be aware of the study so that every CSDH patient will be considered for inclusion in the study. During a kick-off meeting, clinical investigators and study support staff at each study center will be trained in communication with potential study participants and their relatives, documentation, including screening protocols, and standard operating procedures established for study purposes. Potential study participants will be recruited through conference presentations and journal articles.

## Assignment of interventions: allocation

### Sequence generation {16a}, concealment mechanism {16b}, and implementation {16c}

After the neurosurgeon has checked the inclusion and exclusion criteria, the participants are assigned to treatment with tranexamic acid or embolization in a 1:1 ratio in each study center by means of block randomization after they have given their informed consent and the baseline data have been recorded.

If, in the course of the randomized group allocation, it will be apparent that there are exclusion criteria for inclusion in the test group in the sense of contraindications for the administration of tranexamic acid, or if it will be determine during the procedure that vascular anomalies prevent embolization in patients randomized to the control group, a crossover to the corresponding comparison group is possible after prior further clarification.

## Assignment of interventions: blinding

### Who will be blinded {17a}

Due to the study design, which compares a drug therapy with an interventional procedure, neither the patients nor the physicians can be blinded. In addition, the embolizate used in the embolization of the patients treated in the control group will always be visible in the imaging control examinations in both MRI and CT, so that neither the radiologist nor the neurosurgeon can be meaningfully blinded here.

### Procedure for unblinding if needed {17b}

Not applicable as it is not a blinded study.

## Data collection and management

### Plans for assessment and collection of outcomes {18a} and data management {19}

**Fig. 2:**
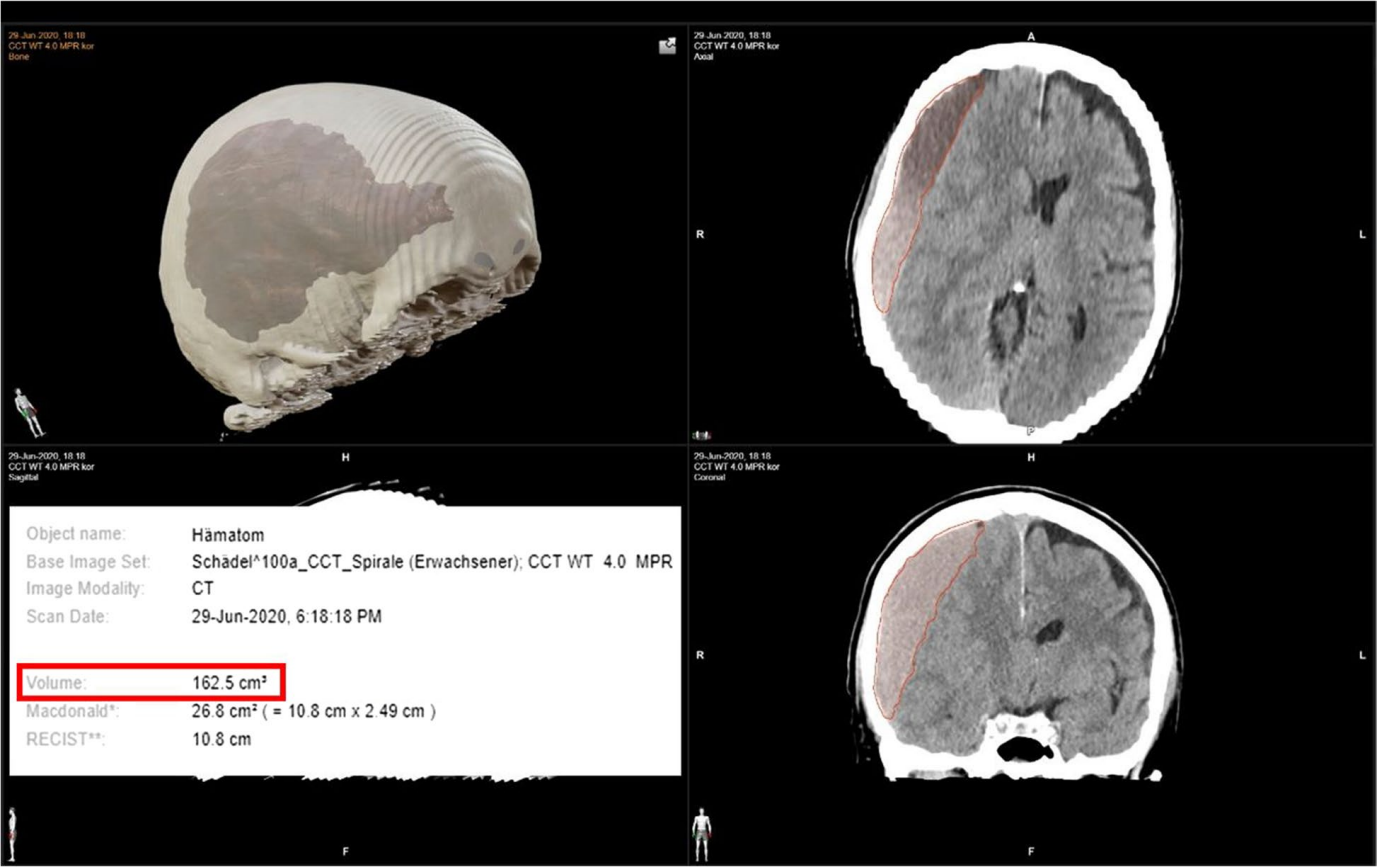
Preoperative volume measurement of a right-sided hemispheric chronic subdural hematoma using the CT data set

Neurological examination results (NIHSS and mRS) at baseline and at each follow-up time point will be recorded and securely stored by the clinical investigators and supporting study personnel in the form of paper data sheets. The size and extent of the CSDH will be measured using CT or MRI scans without contrast. The volume is measured using a medical image viewing program. (Fig. 2) Follow-up is carried out as shown in Table 1 at 1 week, 1, 2, and 3 months postoperatively after relief of the CSDH during outpatient follow-up visits.

The following intervention-associated complications will be documented as part of the study:

- Vascular dissections caused by the guidewire
- Dislocation of embolized material
- Ischemia caused by vascular occlusion during the intervention
- Inflammation or abscesses at the puncture site
- Vascular fistula in the area of the puncture site
- Retinal blindness
- Epidural hematomas after perforation of the meningeal artery
- Allergic reactions to the contrast medium
- Acute renal failure

The following complications or adverse events associated with the use of tranexamic acid will be documented:

- Intolerance reactions
- Pulmonary embolisms
- Venous thrombosis
- Epileptic seizures
- Myocardial infarctions
- Cerebral ischemic infarctions
- Acute renal failure

All research data, including baseline data (NIHSS, mRS, hematoma volume, patient demographics and characteristics) prior to surgery, will be collected anonymously in an electronic data collection form at each study center. The PI of the trial summarizes these for all centers and exports data for statistical analysis. All investigators will be trained to perform outcome measurements and will be introduced to the data management prior to recruitment of the first patient.

### Plans to promote participant retention and complete follow‐up {18b}

Participants will be informed about the study’s objective and relevance. The importance of completing the treatment and the follow-up evaluation, both for the study’s quality and for their personal chance to benefit from the therapy, will be emphasized. Participating patients are consciously informed that there are no personal benefits to take part in the study. Nevertheless, it is also explained that there is scientific evidence that patients who are treated as part of a clinical trial can obtain better results than patients who are treated under routine conditions.

Study follow-up is performed at 1 week and 1, 2, and 3 month (± 1 week) after evacuation of the hematoma with an outpatient clinic visit and CT or MRI scans. During these visits, all outcome measurements are performed by one of the researchers. To maximize participant retention, this study follow-up is parallel to the regular follow-up in the outpatient clinic (standard treatment). In addition, appointments for follow-up examinations are made as soon as the patient is discharged from inpatient treatment in order to increase compliance with participation.

### Confidentiality {27}

All persons involved in the study are bound to confidentiality. All data is collected and analyzed in pseudonymous form. Extensive measures are taken to protect the data, especially personal data, from access by third parties. Local therapy files and study data are kept in locked cabinets and are only accessible to the study staff and the respective treating therapists, who are subject to legal confidentiality.

All treatment cases are managed using identification codes or anonymized registration numbers. The semantics of the patient coding is specified by the Principal Investigator (PI) of the trial and consists of the study center number (two digits) and a consecutive number within the study center (three digits). The correspondence table of anonymization codes and names as well as the consent form with the names are kept strictly in a separate, lockable document storage and managed by the principal investigator of the respective center. The pseudonymous research data will only be shared after a data transfer agreement is signed. If participants withdraw their consent to the study, they will be asked about the reasons for their early withdrawal. The data collected to date will continue to be used and analyzed as part of the study unless the participants withdraw their consent to the processing of their data.

### Plans for collection, laboratory evaluation and storage of biological specimens for genetic or molecular analysis in this trial/future use {33}

Not applicable as no biological specimens are collected as part of this trial.

## Statistical methods

### Statistical methods for primary and secondary outcomes {20a}

The baseline data and outcome parameters are subjected to a simple descriptive data analysis (median, minimum, maximum and standard deviation in each case; mean only for metrically scaled characteristics).

For difference tests for nominal variables (gender, use of anticoagulation prior to surgery, occurrence of complications due to adjuvant therapy procedures, need for revision surgery), the Pearson chi-squared test is used.

For difference tests of ordinally scaled measured variables (NIHSS, mRS) within the respective groups at different points in time (linked samples), the data collected is tested for normal distribution using the Shapiro–Wilk test. In the case of a parametric distribution, the *t*-test is used for linked samples. In the case of a non-parametric distribution, the Wilcoxon test is used for connected samples (for two-group tests).

For the bivariate dependency analysis of the ordinal scaled characteristics between the two groups at the same point in time, the *t*-test for unconnected samples is used for a parametric distribution and the Mann–Whitney *U*-test for a non-parametric distribution after applying the Shapiro–Wilk test.

For metric variables (hematoma volume), which are subject to a normal distribution, the *t*-test for dependent samples is used to analyze the respective groups at different points in time. In the case of a non-parametric distribution, the Wilcox sign-rank test is used for the analysis.

For the bivariate dependency analysis of the metric characteristics between the two groups at the same point in time, after applying the Shapiro–Wilk test, the *t*-test for unrelated samples is used for a parametric distribution and the Mann–Whitney *U*-test for a non-parametric distribution.

In addition, a logistic regression is tested with the revision rate as the dependent variable and the treatment groups and baseline data as independent variables.

Furthermore, a correlation analysis of the relationship between the postoperative volume size and the revision rate as well as the postoperative neurological assessment and the revision rate is performed, in each case for mixed scaling pairs with the chi-squared test. The respective singular correlation analysis is followed by an analysis of the existence of a partial correlation between the two parameters in relation to the revision rate.

The significance level is set at *p* < 0.05 in each case.

The statistical analysis is performed with SPSS.

### Interim analyses {21b}

There are no interim analyses planned. Given that both adjuvant therapy procedures after relief of a chronic subdural hematoma medication with TXA and embolization of the MMA are already validated in clinical practice and in the literature, there is no reason to anticipate exceptionally poor outcomes or unexpected complications. Furthermore, reaching a statistically significant difference between the groups before the planned end of the study is unlikely with the anticipated cohort size.

### Methods for additional analyses (e.g., subgroup analyses) {20b}

A subgroup analysis is not yet planned but will be added if necessary.

### Methods in analysis to handle protocol nonadherence and any statistical methods to handle missing data {20c}

Missing data will be avoided as much as possible by planning scheduled visits well in advance. If a significant amount of data is missing for any of the primary endpoints or other analyses, we will conduct exploratory analyses to assess the impact of the missing data, according to the EMA-guidelines [4]. Depending on the amount of missing data, it will be assessed whether missing covariates can be replaced by multiple imputation with predictive mean adjustment.

### Plans to give access to the full protocol, participant‐level data and statistical code {31c}

The trial was registered on 02 Feb 2024 (http://www.drks.de/DRKS00033515) with the German Clinical Trials Registry (Deutsches Register Klinischer Studien [DRKS]) as a primary registry of the WHO International Clinical Trials Registry Platform (ICTRP; https://trialsearch.who.int/). In addition, data sets.

analyzed in the current study and the statistical code are available upon reasonable request from the corresponding author, as is the full protocol.

## Oversight and monitoring

### Composition of the coordinating center and trial steering committee {5 d}

This study is conducted as a multicenter study. The coordinating team consists of the principal investigator and the study team, which includes the participating physicians from the leading center (Department of Neurosurgery at the Bundeswehrkrankenhaus Ulm), the physicians responsible for the study at each participating center, and the documentation assistants from the initiating center. This group meets regularly (once a quarter) to discuss the progress of the study and any problems that may arise, and to ensure compliance with the study protocol. They support the study on a day-to-day basis. The principal investigator works with the study team to solve any recruitment problems that may arise. In addition, the principal investigator ensures that new study staff receive appropriate training and instruction. Each co-principal investigator of a participating study center is thoroughly informed about the study protocol in a kick-off meeting before the start of study participation. In addition, meetings with all co-investigators will take place at regular intervals to discuss the progress of the study.

The steering committee of the study consists of the PI, experts from the participating departments of neurosurgery and neuroradiology, and the ethics committee of the coordinating center. Regular meetings of the steering committee are planned every 6 months.

### Composition of the data monitoring committee, its role and reporting structure {21a}

Safety of the trial participants will be ensured by an institutional panel consists of the ethical commission, two clinical experts who are independent of the trial and two members of the study team. They monitor serious adverse events (SAEs), the ethical conduct of this study, ensuring that the trial is implemented according to the protocol and that data are collected appropriately.

### Adverse event reporting and harms {22}

Adverse events are defined as any undesirable experience occurring to a subject during the study, whether or not considered related to the investigational product (TXA) or investigational intervention (embolization). After participants have provided consent and been enrolled in the study, adverse events or complications of the treatment, will be documented and recorded until the end of the study period. Any serious adverse event that occurs in this trial will be reported to the principal investigator (PI). The PI must decide whether the participant can continue to participate in the study or whether he or she has to be excluded. The study team in every participating center will take responsibility for the treatment of the trails related adverse events or complications. The PI of the study will consider the continuation or discontinuation of the research under the following circumstances:

- ◦ If a serious adverse event or disease report suggests that continuing the study poses safety concerns
- ◦ If recruiting participants for the study becomes challenging, making it difficult to achieve the planned number of cases
- ◦ When significant new information regarding the quality, safety, or efficacy of the medication (TXA) and interventional procedure (embolization) is obtained

### Frequency and plans for auditing trial conduct {23}

The coordinating team will meet quarterly to discuss study progress, address any issues that may arise, and ensure compliance with the study protocol. The study steering committee, which includes the ethics committee of the leading study center, will meet quarterly. A data monitoring committee was not considered for this study because the intervention of this study is considered low risk.

### Plans for communicating important protocol amendments to relevant parties (e.g., trial participants, ethical committees) {25}

All relevant changes that may affect the conduct of the study, the study protocol or the safety of the participants are initially reported to the study sponsor. The change report to the participating centers is made via the PI, who sends the amended study protocol to the study officers at the respective centers, who add the updated protocol to the Investigator Site File. The amendment is reported to the ethics committees of the leading center and the participating centers in the form of a formal amendment application. In the case of changes that directly affect participants, the patient information sheets, and informed consent forms are adapted (which also will be added to the Investigator Site File) and all study participants are informed. Protocol amendments are updated in the German Clinical Trials Register as they occur. In general, any deviations from the trial protocol will be documented using a breach report form.

### Dissemination plans {31a}

Upon completion of the data collection, a comprehensive biometric report will be prepared by the principal investigator summarizing the key study findings and made available to the participating centers. Finally, the results will be discussed at a meeting of the participating researchers. In this context, a comprehensive publication and dissemination plan will be elaborated, including the publication of research results in the form of conference presentations and the publication of peer-reviewed research articles. In addition, a summary of the results and information on where they can be accessed will be published in the DRKS and thus made available to all interested parties.

## Discussion

We have described the protocol for a prospective, multicenter, randomized study comparing the influence of adjuvant drug therapy with tranexamic acid and additional endovascular embolization of the MMA after primary surgically relieved chronic subdural hematomas in terms of hematoma volume reduction, revision rate and safety of the procedures. The current study results indicate that both procedures can reduce the postoperative revision rate in particular. However, a randomized controlled trial is urgently needed to evaluate the risk–benefit ratio of additional embolization of the MMA compared to adjuvant drug therapy with TXA and, in this context, to identify patient entities with a comparable risk–benefit profile for whom one or the other adjuvant therapy is the appropriate treatment model. The prospective, multi-center design of this study promises a high level of evidence in this context.

### Trial status

This manuscript is based on trial protocol version 2.1, dated 25 April 2024. Recruitment at the leading center started on 01 May 2024. The patient recruitment has also started at a second study center. A further participating study center has recently been acquired. Recruitment has not started here. Several other potential study sites have been contacted. The end of the recruitment phase depends on the total number of participating study centers and is scheduled for the beginning of 2026 if a total of 5–8 sites are involved.

## Supplementary Information

Supplementary Material 1

## Data Availability

Until the publication of the study results, only the principal investigator and participating institutions have access to the data set. Non-identifiable data sets can be made available by the principal investigator upon reasonable request and after publication of study results. There are no contractual agreements.

## Abbreviations

cMRI: Cranial magnetic resonance imaging
CSDH: Chronic subdural hematoma
CT: Computed tomography
EMA: European Medicines Agency
DKRS: German Clinical Trials Registry
DSA: Digital subtraction angiography
MMA: Middle meningeal artery
mRS: Modified Rankin Scale
MRI: Magnetic resonance imaging
NIHSS: The National Institutes of Health Stroke Scale
PI: Principal investigator
SAEs: Serious adverse events
SPSS: Statistical Package für Social Sciences (IBM®)
TABASCO: Tranexamic acid vs. embolization of the meningeal artery as an adjunctive therapeutic regime to reduce the recurrence rate after surgical relief of chronic subdural hematomas
TXA: Tranexamic acid
